# Increased expression of CYP17A1 indicates an effective targeting of the androgen receptor axis in castration resistant prostate cancer (CRPC)

**DOI:** 10.1186/2193-1801-3-574

**Published:** 2014-10-01

**Authors:** Felix Bremmer, Hubertus Jarry, Arne Strauß, Carl Ludwig Behnes, Lutz Trojan, Paul Thelen

**Affiliations:** Institute of Pathology, University Medical Center, University of Göttingen, Robert-Koch-Str. 40, 37075 Göttingen, Germany; University Medical Center, University of Göttingen, Göttingen, Germany; Department of Urology, University Medical Center, University of Göttingen, Robert-Koch-Str. 40, 37075 Göttingen, Germany

**Keywords:** Abiraterone acetate, CYP17A1, CRPC, Androgen receptor, IGF-2

## Abstract

Recent breakthrough therapies targeting androgen receptor signalling in castration resistant prostate cancer (CRPC) involve multifunctional androgen receptor (AR) blockade and exhaustive androgen deprivation. Nevertheless, limitations to an enduring effectiveness of new drugs are anticipated in resistance mechanisms occurring under such treatments.

In this study we used CRPC cell models VCaP and LNCaP as well as AR-negative PC-3- and non-neoplastic epithelial BPH-1-cells treated with 5, 10 or 25 μmol/L abiraterone hydrolyzed from abiraterone acetate (AA). The origin of CYP17A1 up-regulation under AA treatment was investigated in CRPC cell models by qRT-PCR and western-blot procedures.

AA treatments of AR positive CRPC cell models led to decreased expression of androgen regulated genes such as PSA. In these cells diminished expression of androgen regulated genes was accompanied by an up-regulation of CYP17A1 expression within short-term treatments. No such effects became evident in AR-negative PC-3 cells. AR directed siRNA (siAR) used in VCaP cells significantly reduced mRNA expression and AR protein abundance. Such interference with AR signalling in the absence of abiraterone acetate also caused a marked up-regulation of CYP17A1 expression. Down-regulation of androgen regulated genes occurs in spite of an elevated expression of CYP17A1, the very target enzyme for this drug. CYP17A1 up-regulation already takes place within such short treatments with AA and does not require adaptation events over several cell cycles. CYP17A1 is also up-regulated in the absence of AA when AR signalling is physically eliminated by siAR.

These results reveal an immediate counter-regulation of CYP17A1 expression whenever AR-signalling is inhibited adequately but not a persisting adaptation yielding drug resistance.

**Electronic supplementary material:**

The online version of this article (doi:10.1186/2193-1801-3-574) contains supplementary material, which is available to authorized users.

## Background

Prostate cancer is the second leading cause of cancer death from the most frequently diagnosed malignancy in males in the USA. As well in Europe prostate cancer accounts for 9% of total cancer-related deaths. Standard treatments for localized prostate cancer are radical surgery, radiation therapy or active surveillance (Ferlay et al. 2007; Jemal et al. 2011). However conventional therapies may fail as almost one-third of patients who undergo local treatment will experience relapse and will receive androgen deprivation therapy (Kantoff and Mohler 2013).

Within the last three years two alternative anti-androgen strategies reached clinical application offering new options in both pre- and post-chemotherapy setting which emphasises the androgen receptor as sustained therapy target in prostate cancer. Chemotherapy often succeeds temporarily following therapy failure of conventional hormone therapies. New drugs including abiraterone acetate (AA) and enzalutamide, effectively target the androgen pathway to arrest aberrant signalling even after multiple therapies. The mode of action of these new compounds is either inhibition of androgen synthesis at the CYP17A1 enzyme covering also adrenal and intracrine androgen sources, or a full androgen receptor (AR) blockade antagonizes the AR function by directly binding this transcription factor with high affinity. Nevertheless, clinical success of these novel drugs is not enduring in all cases. Recent phase 3 trials revealed median time to PSA progression of about eight months for both drugs and in sequential treatments of enzalutamide and AA cross resistances have to be considered (Schrader et al. 2013). Whilst the molecular mechanisms for cross resistances occurring after sequential use of androgen ablation and androgen receptor blockade are still elusive, the alterations leading to individual therapy resistance have been addressed in more detail. The resistance mechanisms can be distinguished between AR amplification/overexpression including alternative AR splice variants (e.g. VCaP) and gain-of-function AR mutations in the ligand binding domain (e.g. LNCaP) associated with anti-androgen treatments notably flutamide or bicalutamide (Knudsen and Penning 2010; Waltering et al. 2012). Even for the second-generation anti-androgen enzalutamide a ligand binding domain mutation has been identified which converts the drug from an AR antagonist to an AR agonist. However, this mutation was identified in a mutagenesis screen in cell models (Balbas et al. 2013).

Recent considerations of actual therapy resistance include newly acquired mechanisms within the AR-axis under the individual treatment and do not necessarily require ultimate castration resistance as in AR-negative cell models of prostate cancer (Feldman and Feldman 2001). In case of CYP17A1 inhibitor treatments an increased expression of CYP17A1 thereafter was assumed as a potential mechanism and dose escalation or combined treatments with enzalutamide were suggested to cope with this effect (Cai et al. 2011; Richards et al. 2012; Mostaghel et al. 2011). In the present study we analyse the causation of CYP17A1 up-regulation and discuss how this phenomenon qualifies for a therapy resistance mechanism in distinct prostate and CRPC cell models varying in AR status.

## Methods

### Culture cell lines and treatment

Human prostate cancer cell line VCaP was purchased from ATCC, Wesel, Germany and kept in Phenol red-free Gibco® DMEM lot # 1089200 (Life Technologies GmbH, Darmstadt, Germany) supplemented with 2% sodium pyruvate and 10% foetal calf serum (PAA, Cölbe, Germany). The other cell lines LNCaP, PC-3 and BPH-1 from permanent local stocking were certified to proof origin (Fuessel et al. 2013). Cells were incubated at 37 °C and 5% CO_2_ in a humidified incubator. Cells and supernatants were harvested for RNA and protein extraction or used for ELISA assays. PSA release into cell culture media was measured with PSA-ELISA EIA-3719, (DRG Instruments, Marburg, Germany). This study did not require an approval from an ethics committee and the local animal protection committee.

Abiraterone acetate (Janssen Cilag, Neuss, Germany) was used for treatments in 5, 10 or 25 μmol/L concentration performed for 24 hours. To obtain the active drug hydroxy-abiraterone (AA), abiraterone acetate was hydrolysed in 95% ethanol prior to use (Soifer et al. 2012).

### siRNA transfection

The VCaP cells were transfected with siRNAs against the androgen receptor (Riboxx Radebeul, Germany). To select for best AR interference four different sequences were used (Table 1). We chose the siRNA sequence ARex7 which had the highest down-regulation to 16% of regular expression (Additional file 1: Figure S1). Before transfection for 24 h, 10^5^ tumour cells were plated in six well plates for 48 h in DMEM. The transfection medium consists of 10 μl from a 20 μmol/l oligonucleotide stock solution, 5 μl Oligofectamine reagent, 185 μl Opti-MEM according to the manufacturer recommendation (Life Technologies GmbH). Luciferase (LUC) siRNA was used for control transfections (Eurogentec, Cologne, Germany).

**Table 1:** **Different sequences of siRNA against the AR**

	Sequence	Sense strand
ARex2	5′-UUGAAGAAGACCUUGCAGCCCCC-3′	5′-GGGGGCUGCAAGGUCUUCUUCAA-3′
ARex2/3	5′-ACUUCUGUUUCCCUUCAGCCCCC-3′	5′-GGGGGCUGAAGGGAAACAGAAGU-3′
ARex4	5′-AUUACCAAGUUUCUUCAGCCCCC-3′	5′-GGGGGCUGAAGAAACUUGGUAAU-3′
ARex7	5′-AUCUCUGCCAUCAUUUCCGCCCCC-3′	5′-GGGGGCGGAAAUGAUGGCAGAGAU-3′

### mRNA-expression analysis

mRNA-expression of PSA, TMPRSS2-ERG, CYP17A1, AKR1C3, AR and IGF-2 were analysed by qRT-PCR. Total cellular RNA from cultured cells was extracted with the Quick-RNA™MiniPrep (Zymo Research, Freiburg, Germany). Total RNA integrity and quantity were assessed on an Agilent Bioanalyzer 2100 with a RNA 6000 Nano LabChipKit (Agilent Technologies, Waldbronn, Germany). Reverse transcription of 500 ng total cellular (tc) RNA was performed with random hexamer primers and an Omniscript RT Kit (QIAGEN, Hilden, Germany). Expression analyses were processed on an iCycler iQ real time detection system (BIORAD, Munich, Germany) with SsoFast EvaGreen supermix. The 20 μl reaction from the kit was supplemented with 2 μl cDNA, 0.6 μM gene-specific primers (IBA, Göttingen, Germany). Primers were designed by Primer 3 and PCR efficiency was assessed as previously described (Stettner et al. 2007).

### Western blot analysis

Protein expression was measured by western blot analysis with androgen receptor antibody Ab-2 (Cat. # RB-1358, Lab Visions Corp. Fremond, CA, USA) and CYP17A1 antibody (Cat. 14447-1-AP, protein tech, Manchester, United Kingdom) where α-tubulin was used as loading control detected with a monoclonal anti-α-tubulin (clone B-5-1-2, # T 5168, Sigma, St. Louis, MO, USA). Cells were homogenized with Pierce® RIPA buffer (Thermo Scientific, Rockford, IL, USA). Novex® precast gels (Life Technologies, Darmstadt, Germany) were used for electrophoresis followed by electro-transfer onto Protran® nitrocellulose membranes (Whatman GmbH, Dassel, Germany) from where protein-bound membrane was hybridized with antibodies. For visualization, we used Western Lightning® Plus-ECL (Perkin Elmer, Waltham, MA, USA), rabbit secondary antibodies (P 0448 Dako, Glostrup, Denmark) and ProteinSimple FluorChem E (BioZym Hess. Oldendorf, Germany). For quantification of band intensity Image J (http://imagej.nih.gov/ij/) was used.

### Statistical analyses

Statistical calculation, mean +/-SD and P values were carried out with GraphPad Prism software version 5.0 using the unpaired nonparametric *t* test at 95% confidence interval considered statistically significant (* = p < 0.05, ** = p < 0.005 and *** = p < 0.0005).

## Results

### Treatment of VCaP and LNCaP Cells with AA

**Figure 1:**
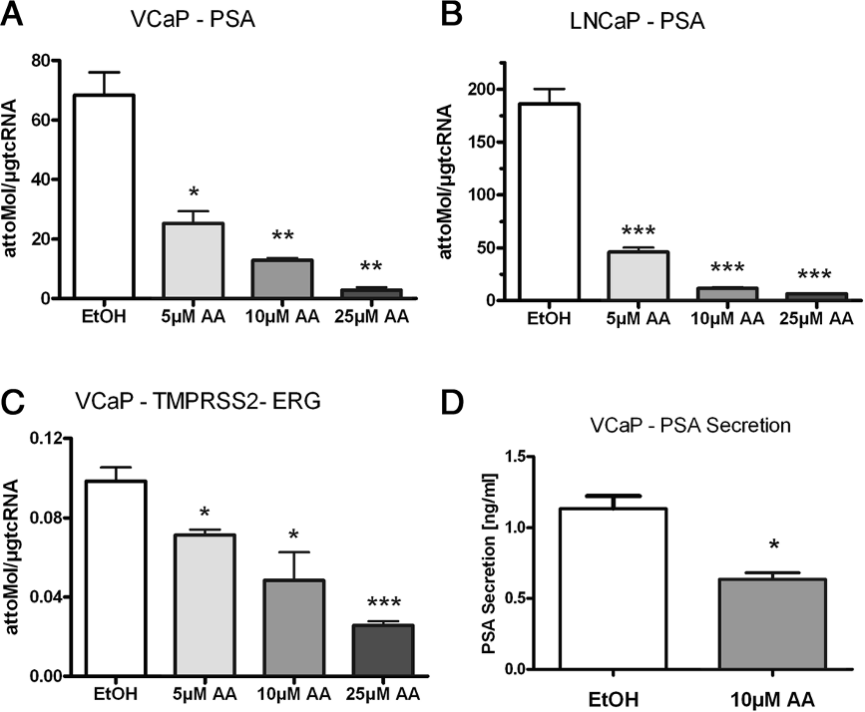
**Expression profile of PSA and TMPRSS2-ERG: VCaP and LNCaP showing decreased PSA and TMPRSS2-ERG (VCaP) mRNA expression after treatment with with 5, 10 or 25 μM AA (A-C).** In addition treatment with 10 μM AA leads to a decreased PSA secretion in VCaP **(D)**; (* = p < 0.05, ** = p < 0.005 and *** = p < 0.0005).

**Figure 2:**
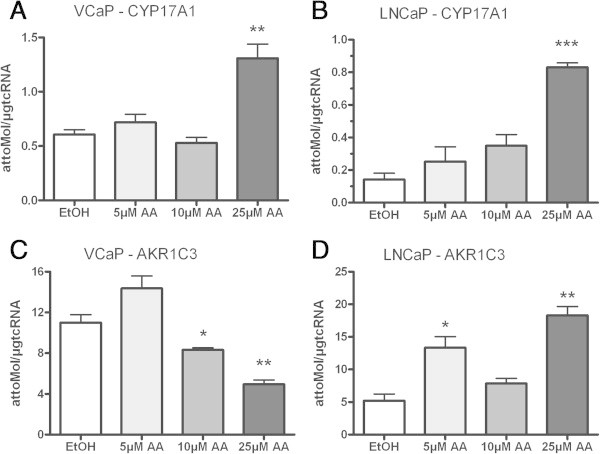
**Expression profile of CYP17A1 and AKR1C3: CYP17A1 expression level is significantly increased in VCaP and LNCaP after treatment with 25 μm AA (A + B).** In addition AKR1C3 mRNA is decreased in VCaP and increased in LNCaP cell line **(C + D)**; (*= p < 0.05, **= p < 0.005 and ***= p < 0.0005).

**Figure 3:**
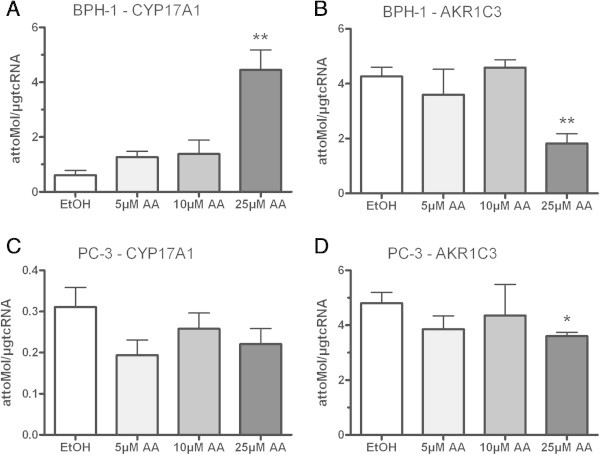
**CYP17A1 and AKR1C3 expression in BPH-1 and PC3: increased CYP17A1 and decreased AKR1C3 mRNA in BPH-1 after treatment with 25 μM AA (A + B).** In PC-3 no significant effects after AA treatment in CYP17A1 and AKR1C3 mRNA expression could be seen **(C + D)**; (*= p < 0.05, **= p < 0.005 and ***= p < 0.0005).

CRPC cell line VCaP with overexpressed AR and LNCaP with a gain-of-function AR mutation were treated with 5, 10 or 25 μmol/L AA. In both cell lines PSA mRNA expression decreased after treatment with AA (Figure 1A + B). The same effect was found for TMPRSS2-ERG expression in the VCaP cell line (Figure 1C). According to the PSA mRNA expression the PSA secretion also decreased (Figure 1D). In the same experiments we looked for the expression profile of CYP17A1 and AKR1C3. Concomitantly with the detected decrease of androgen regulated genes PSA and TMPRSS2-ERG, in both cell lines the CYP17A1 expression level significantly increased after treatment with 25 μmol/L AA (Figure 2A + B). In contrast, the downstream enzyme in testosterone biosynthesis, AKR1C3, reveals decreased mRNA expression levels under such treatments in VCaP but not in LNCaP cells (Figure 2C + D). Counter-regulated CYP17A1 and AKR1C3 mRNA levels were also found in the non-neoplastic cell line BPH-1 (Figure 3A + B) indicating an association of this phenomenon with a non-mutated ligand binding domain of the AR. In contrast to all other cell models the AR-negative, malignant tumour cell line PC-3 treated with AA does not show any significant effects on CYP17A1 and AKR1C3 expression (Figure 3C + D).

### Targeting the AR in VCaP cells with siRNA elicits CYP17A1-up-regulation in absence of AA

**Figure 4:**
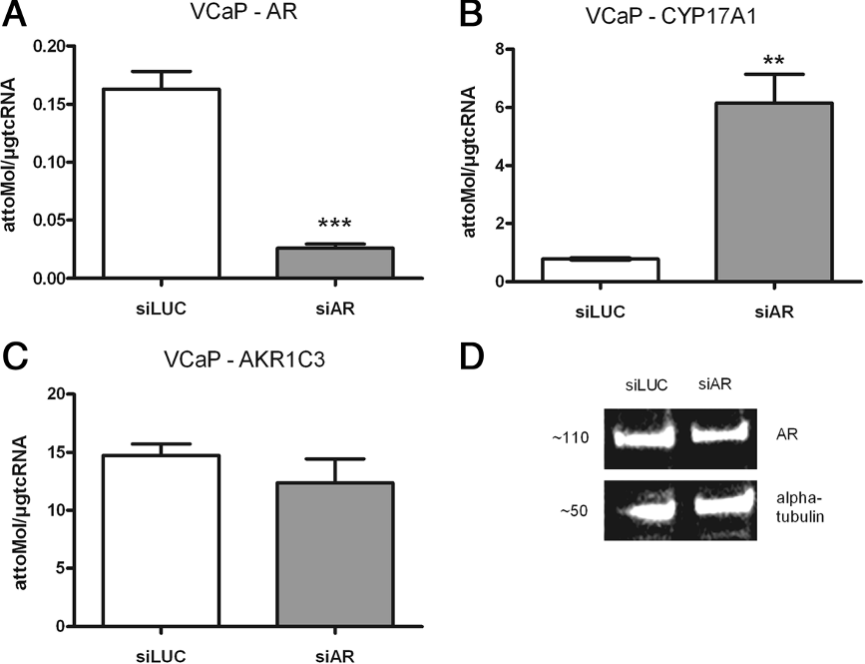
**AR targeting siRNA in VCaP: siRNA against AR significantly reduced AR expression and an up-regulation of CYP17A1 expression in the absence of AA.** No such effect is seen in AKR1C3 mRNA expression levels **(A-D)**; (*= p < 0.05, **= p < 0.005 and ***= p < 0.0005).

A siRNA against AR transcripts was used to elucidate the relevance of AR signalling to the effects on CYP17A1 and AKR1C3 expression in the absence of the compound AA. AR directed siRNA significantly reduced AR mRNA-expression and is also seen in western blot analysis (Figure 4A), albeit high AR expression is persistent in VCaP cells. However, this interference with AR expression in the absence of AA also caused an up-regulation of CYP17A1 expression in VCaP cells (Figure 4B), but had only marginal effects on AKR1C3 expression (Figure 4C). Therefore, CYP17A1 up-regulation is not dependent on AA presence and is based upon AR impairment.

### CYP17A1 enzyme inhibition and androgen receptor expression under AA treatment

**Figure 5:**
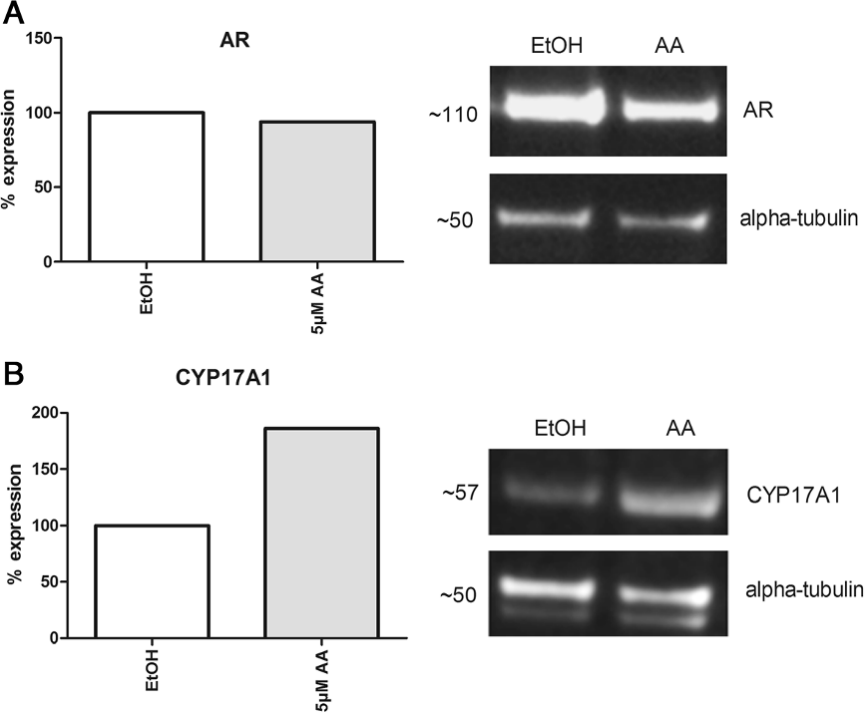
**Western Blot analysis of the AR and CYP17A1 after AA treatment with 5 μmol/L.** In western blot analyses no significant changes were detected on the AR protein level **(A)**, whereas CYP17A1 protein expression increased markedly **(B)**.

To elucidate by which function in AR signaling, receptor status or androgen synthesis, androgen regulated genes are affected, western blot analysis for the AR and CYP17A1 were performed. In these experiments VCaP cells were treated with 5 μmol/L AA. No significant changes were detected on the AR protein level calculated from western blot analyses (Figure 5A + C), whereas in the same experiment CYP17A1 protein expression increased markedly (Figure 5B + C). This experiment excludes a loss of AR expression as an explanation for diminished androgen receptor signaling and implies a full arrest of CYP17A1-mediated androgen synthesis by AA even when the enzyme was overexpressed.

### Correlation of AA treatment effects on steroidogenesis and IGF-2 expression

**Figure 6:**
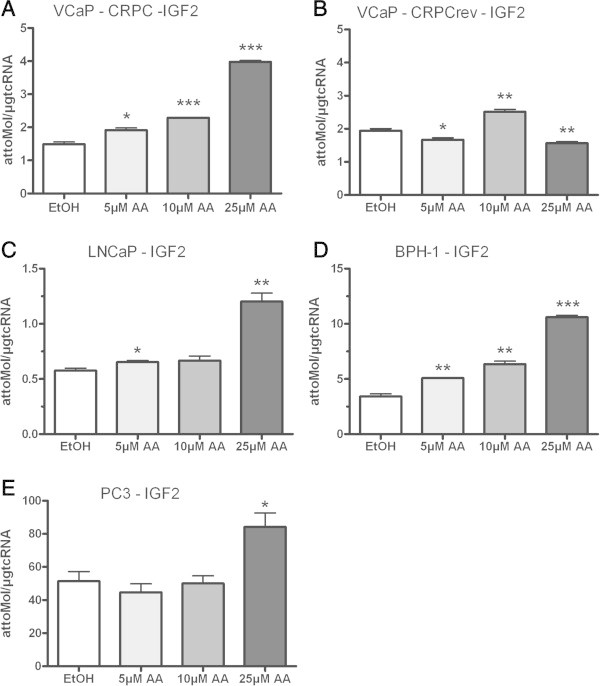
**Correlation of AA treatment on IGF-2 expression: IGF-2 is up-regulated in VCaP-CRPC, LNCaP, BPH-1 and PC3 after treatment with 25 μm AA (A, C-E).** In contrast IGF-2 expression was not elevated after treatments of increasing AA concentrations in VCaP-CRPCrev **(B)**; (*= p < 0.05, **= p < 0.005 and ***= p < 0.0005).

As the entire IGF-axis has significant influence on mechanisms of castration resistance, IGF-2 is involved in pathways which can induce *de novo* steroidogenesis. Therefore, we investigated the influence of AA on IGF-2 expression in our different prostate cell models. Concomitant with decreased expression of androgen regulated genes despite CYP17A1 up-regulation, IGF-2 expression was also up-regulated with increasing AA concentrations in VCaP CRPC cells (Figure 6A). We also established a VCaP variant reverted to androgen sensitivity termed VCaP CRPCrev. These cells growing steadily under 1 nmol/L testosterone showed a similar basic level of IGF-2 expression which was not elevated with treatments of increasing AA concentrations (Figure 6B). This indicates a marker function of IGF-2 for androgen deprivation. Under these conditions LNCaP only showed an increased IGF-2 expression after treatments of 25 μmol/L AA (Figure 6C). In contrast the non-neoplastic BPH-1 cells revealed a stepwise increase of IGF-2 expression (Figure 6D) similar to VCAP CRPC cells (Figure 6A). Interestingly, also AR negative malignant prostate cancer cells (PC-3) exhibit an increased IGF-2 expression like LNCaP at higher AA concentration (Figure 6E). In these PC-3 cells this increase of IGF-2 expression in contrast to AR expressing cells is not associated with an increase of CYP17A1 expression (Figure 3C).

## Discussion

The huge progress made in the past few years to treat CRPC with hormonal manipulation or AR blockade caused confidence in prostate cancer therapy but may be compromised by resistance mechanisms acquired under these novel treatments (Yuan et al. 2013). Therapy resistance may result of ultimate castration resistance or other undefined escape mechanisms. Such therapy resistances developing under treatment and hence selective pressure on tumour cells are distinguishable from mechanisms responsible for initial therapy failure in non-responding patients. One obvious explanation for therapy induced resistance is tumour growth of androgen independent cell clones as evident in tumour cell models (Balbas et al. 2013; Sharma et al. 2010). Furthermore, therapy resistance developed under AA treatments has been explained by treatment-induced selection of tumour cells with elevated intratumoural expression of CYP17A1. Indeed in a study by Cai et al. an increased mRNA becomes evident in VCaP xenograft tumour bearing mice after long-term AA treatments, whereas only one in four xenografts had increased CYP17A1 expression in short term treatments with the drug (Cai et al. 2011). From this phenomenon the authors infer a *de novo* intratumoural steroid synthesis and a mechanism contributing to AR reactivation and resistance to CYP17A1 inhibitors.

In our short term *in vitro* experiments in VCaP cells, however, we found an increased CYP17A1 expression with increasing concentrations of AA within 24 hours. Despite countering CYP17A1 expression androgen regulated genes such as PSA or the fusion transcript TMPRSS2-ERG were down-regulated with increasing AA concentrations, indicating sufficiently suppressed androgen receptor signalling in the presence of the drug. This confirms a still complete inhibition by AA of basal and surplus CYP17A1 enzyme activity under continuous treatment. To exclude an alternative explanation for this finding we calculated AR expression and CYP17A1 expression side-by-side from western blot analyses (Figure 5). Hence, AR expression is not affected by AA treatments with low concentrations of 5 μmol/L whereupon CYP17A1 is elevated then. This lead to the conclusion that an inhibition of androgen signalling in the presence of overexpressed CYP17A1 was not due to altered AR expression.

Based on AR alterations there are two distinct explanations for castration resistance and evolving therapy resistance. One is AR overexpression to retrieve trace amounts of androgens and the other selection for ligand binding domain mutations to utilize a variety of alternative ligands for activation (Balbas et al. 2013).

Although a direct interaction of AA with the AR and a switch to an agonist of a mutated AR has been discussed as mechanism for therapy resistance (Cronauer et al. 2013), our results can all be explained by an exclusive AA effect on steroidogenesis and low affinity to AR. Actually, selection for a gain-of-function mutation in prostate cancer cells is most conceivable when androgens are scarce as under androgen deprivation and in the presence of high-affinity AR ligand. A therapy resistance based upon this principle may only arise under androgen deprivation in combination with an anti-androgen with demonstrable high affinity to the androgen receptor. With an effective binding of a ligand, the incidence of a gain-of-function mutation which turns an anti-androgen into an androgen thusly granted prevalence for an activated mutated AR no longer blocked by the anti-androgen. Therefore, therapy resistance, especially a most menacing anti-androgen withdrawal phenomenon is more comprehensible for anti-androgen therapy than androgen deprivation. In this study we showed that CYP17A1 up-regulation, the presumed resistance mechanism to AA therapy is not restricted to androgen signalling in the *de facto* presence of AA. When androgen signalling is impaired by other means e.g. by RNA interference with AR expression by specific siRNA (Figure 4) the same effect becomes evident. Interestingly, VCaP cells under hydroxyl-flutamide or bicalutamide treatment also showed up-regulated CYP17A1 and down-regulated AKR1C3 expression (Kumagai et al. 2013), we demonstrated for AA treatments (Figure 1). Therefore, CYP17A1 up-regulation obviously is indicative for effective impairment of AR signalling in general not limited to AA treatments and may represent a negative feedback loop (Auchus and Auchus 2012). Although all AA effects on AR positive cells VCaP, LNCaP and BPH-1 appear to be attributed to an inhibitory influence on steroidogenesis, our results from AR knock-down experiments and AR negative PC-3 cells cannot fully exclude the notion of AA binding the AR and partly inhibiting AR-signalling. There are conflicting results from previous studies which either excluded binding of AA to AR or detected direct AA binding to the AR *in vitro* assays despite low affinity as compared with pure antiandrogens (Handratta et al. 2005; Richards et al. 2012).

Our study revealed that a therapy resistance to AA treatments is not satisfactorily explained by sole CYP17A1 up-regulation. In addition to an up-regulation of CYP17A1 expression under AA treatments we also showed an increased expression of IGF-2 in a concentration dependent manner. Recent studies revealed new molecular pathways by which IGF-2 confers androgen independent growth or can ignite the *de novo* steroidogenesis engine and promote molecular events associated with tumour progression evading hormone therapy (Lubik et al. 2013; Comstock and Knudsen 2013). IGF2 can activate the IGF1 receptor or insulin receptor or hybrids of these two receptors to contribute to prostate cancer progression to castration resistance (Lubik et al. 2011). Further studies are warranted to evaluate a switch from steroid hormones to mitogenic peptide hormones such as IGF-2 as response to androgen deprivation and antiandrogen therapy and possible escape mechanism to be targeted selectively in new therapy attempts.

## Conclusions

An up-regulation of CYP17A1 expression under AA treatments is inconsistent with otherwise identified benefits in CRPC therapy. The CYP17A1 up-regulation under AA is not restricted to tumour cells but is elicited when AR signaling is impaired. Therefore, this phenomenon represents an immediate feedback loop upon AR impairment, rather than an acquired, persisting drug resistance mechanism. This means the search for the mechanisms of therapy resistance in CRPC is not over. However, the recent anticipation of cross resistances after various anti-androgen concepts suggests the last resort of therapy resistant tumour cells is narrowed down to only few options. Therefore, further research is warranted to target these escape mechanisms unerringly, when even up-to-date regimens begin to falter.

### Ethical standards

This study did not require an approval from an ethics committee and the local animal protection committee.

## Electronic supplementary material

Additional file 1: Figure S1: Effect of siRNA against the AR: ARex7 downregulation to 16%, ARex2 to 26%, ARex2/3 to 40% and ARex4 to 56%. (TIFF 202 KB)

## Abbreviations

CRPC: Castration resistant prostate cancer
AR: Androgen receptor
AA: Abiraterone acetate
CYP17A1: Cytochrome P450 17A1
IGF-2: Insulin-like growth factor
AKR1C3: Aldoketone reductase
TMPRSS2-ERG: Transmembrane protease, serine 2 ets related gene.
