# Supplementary figures and images for: Increased expression of CYP17A1 indicates an effective targeting of the androgen receptor axis in castration resistant prostate cancer (CRPC)
Source: Springerplus. 2014 Oct 1;3:574. doi: 10.1186/2193-1801-3-574 (PMC4193971; doi:10.1186/2193-1801-3-574)

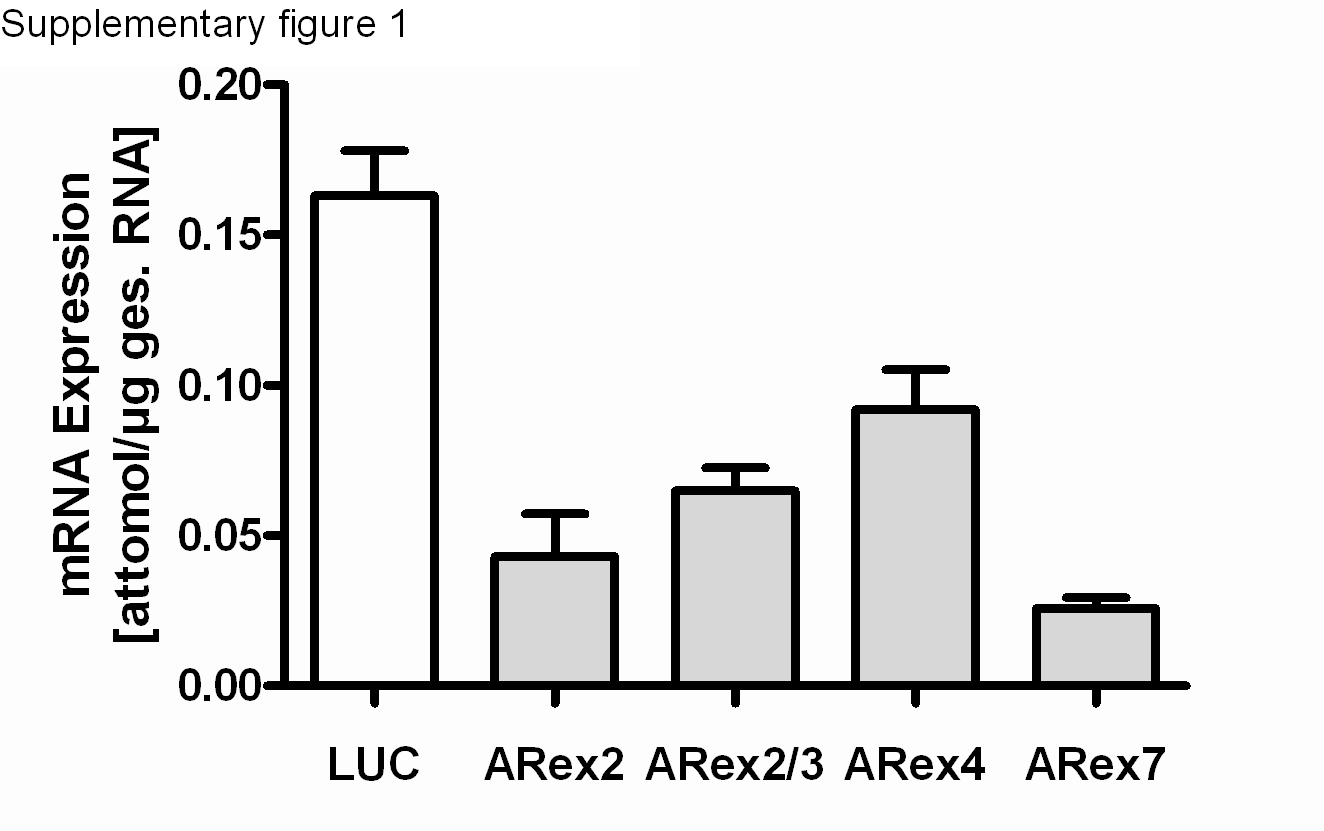

Supplement: Supplementary file 1 — Additional file 1: Figure S1: Effect of siRNA against the AR: ARex7 downregulation to 16%, ARex2 to 26%, ARex2/3 to 40% and ARex4 to 56%. (TIFF 202 KB) [file 40064_2014_1279_MOESM1_ESM.tiff]
